# Development of a Newborn Screening Program for Critical Congenital Heart Disease (CCHD) in Taipei

**DOI:** 10.1371/journal.pone.0153407

**Published:** 2016-04-13

**Authors:** Pei-Chen Tsao, Yu-Shih Shiau, Szu-Hui Chiang, Hui-Chen Ho, Yu-Ling Liu, Yuan-Fang Chung, Li-Ju Lin, Ming-Ren Chen, Jia-Kan Chang, Wen-Jue Soong, Hsiu-Lian Lin, Betau Hwang, Kwang-Jen Hsiao

**Affiliations:** 1 Division of General Pediatrics, Pediatrics Department, Taipei Veterans General Hospital, Taipei, Taiwan; 2 Department of Pediatrics, School of Medicine, National Yang-Ming University, Taipei, Taiwan; 3 Institute of Physiology, School of Medicine, National Yang-Ming University, Taipei, Taiwan; 4 Institute of Emergency and Critical Care Medicine, School of Medicine, National Yang-Ming University, Taipei, Taiwan; 5 Preventive Medicine Foundation, Taipei, Taiwan; 6 Taipei Institute of Pathology, Taipei, Taiwan; 7 Department of Health, Taipei City Government, Taipei, Taiwan; 8 MacKay Children’s Hospital, Taipei, Taiwan; 9 Pediatrics Department, Cheng Hsin General Hospital, Taipei, Taiwan; 10 Taipei City Hospital, Zhongxiao Branch, Taipei, Taiwan; 11 Cardiac Medical Center, Tung’s Taichung Metroharbor Hospital, Taichung, Taiwan; 12 Department of Education and Research, Taipei City Hospital, Taipei, Taiwan; Centers for Disease Control, TAIWAN

## Abstract

**Background:**

Early detection of critical congenital heart disease (CCHD) can significantly reduce morbidity and mortality among newborns. We investigate the feasibility of implementing a community-based newborn CCHD screening program in Taipei.

**Methods:**

Twelve birthing facilities in Taipei participated in a trial screening program between October 1, 2013, and March 31, 2014. Newborns underwent pulse oximetry at 24–36 h old, with probes attached to the right hand and one lower limb. Any screening saturation ≥95% in either extremity, with an absolute difference of ≤3% between the right hand and foot, was accepted as a screening pass. A screening result was considered as a fail if the oxygen saturation was <95% at either probe site, on 3 separate occasions, each separated by 30 min or the first result was <95% at either probe site, and any subsequent oxygen saturation measurement was <90%. Public health nurses would follow up all missed or refused cases.

**Results:**

Of the 6,387 live births, 6,296 newborns (coverage rate: 6,296/6,387 = 98.6%) underwent appropriate pulse oximetry screening. Sixteen newborns (0.25%) were reported to have a failed screening result. Five of these screen positive newborns were confirmed with CCHD; two of them were diagnosed solely attributed to the failed screening results. The false-positive rate was 0.18%. Implementing a 6-month screening program for CCHD produced good case detection rate, while using efficient screening and referral systems.

**Conclusion:**

This program was successful in integrating screening, referral and public health tracking systems. The protocol outlined in this report could provide a community-based model for worldwide implementation.

## Introduction

The overall incidence of congenital heart disease is reported around 7 to 9 per 1000 live births.[1, 2] Of these, one-sixth to one-fourth have critical congenital heart disease (CCHD), which is defined as a severe and life-threatening disease that requires surgical or catheter intervention within the first year of life.[3, 4] Of infants with nonsyndromic CCHD, up to 30% are reported to encountered sudden respiratory distress or circulatory collapse because of late detection.[5, 6] In addition, a previous study found that babies who left hospital with undiagnosed duct-dependent circulations had a relative risk for mortality of up to 16.3 when compared with those diagnosed in hospital.[7] Pulse oximetry is a simple and universal tool for detecting and differentiating preductal and postductal hypoxemia, which is characteristic of most forms of CCHD, but may not be recognized by clinical examination.[8] The major targets of screening by pulse oximetry screening for CCHD include hypoplastic left heart syndrome (HLHS), pulmonary atresia (PS), tetralogy of Fallot (TOF), total anomalous pulmonary venous return (TAPVR), d-transposition of the great arteries (d-TGA), tricuspid atresia (TA), and truncus arteriosus.[9, 10] The secondary targets include coarctation of the aorta (CoA), double-outlet right ventricle (DORV), Ebstein anomaly, interruption of aortic arch (IAA), and single ventricle.[9, 10] Since 2011, pulse oximetry has been recommended as a screening tool for CCHD in newborn,[10] and there has been a growing number of clinical trials on the subject.[11–13] Despite differences among the screening protocols, specifically concerning targets, the time of screening, cut-off values, and probe placement, pulse oximetry has been accepted as a useful screening method.[4, 7, 11]

The prevalence of congenital heart disease (CHD) in Taiwan was estimated around 13.08 per 1000 live births,[14, 15] with approximately one-fifth of these having CCHD. Although advances in surgical techniques as well as pre- and post-operative care have contributed to declining levels of morbidity and mortality due to CHD, delayed detection or failure to diagnose CCHD continues to cause poor neurological outcomes and avoidable deaths. Screening could provide targeted improvements in case detection and treatment. Along similar lines, a newborn screening program has been established for the detection of inborn errors of metabolism within 3 days of birth in Taiwan since 1984, achieving coverage rates of 80% in 1990 and ≥99% since 1996.[16] Based on this screening program, screening for hearing problems among newborns was developed in Taipei City in 2009,[17] and later extended to the entire country in 2012.

In 2013, the Taipei City government, the Prevention Medicine Foundation, and the Taipei Institution of Pathology (TIP) collaborated to develop a community-based screening program that used pulse oximetry to identify newborns with CCHD in Taipei. In this report, we describe the design, implementation, and results of this community-based screening program.

## Methods

**Fig 1:**
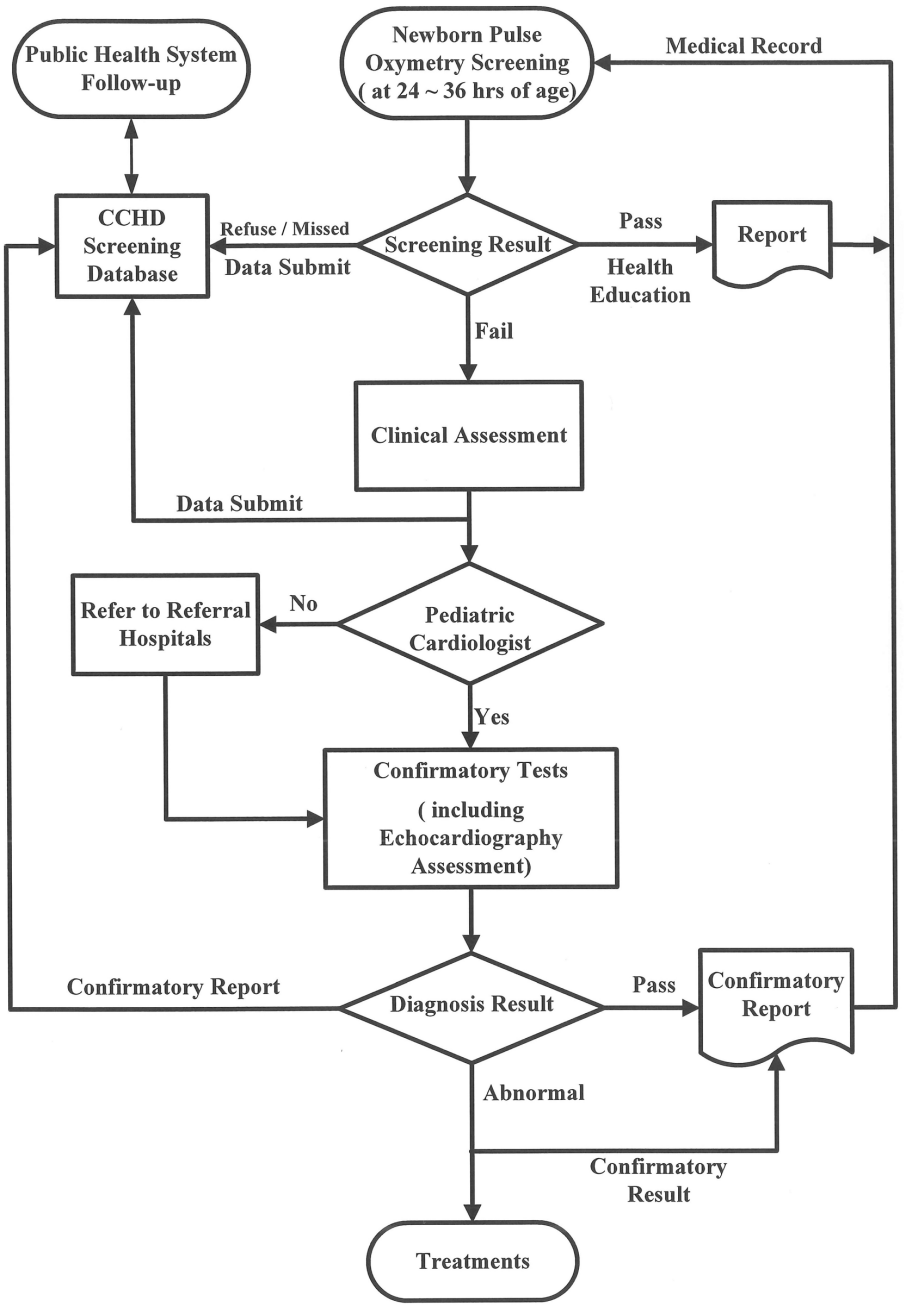
The screening algorithm for CCHD in Taipei. Abbreviation: critical congenital heart disease, CCHD.

This project was reviewed and approved by the Institutional Review Board of Taipei City Hospital. Between October 1, 2013, and March 31, 2014, a community-based program was launched that used pulse oximetry to screen newborns for CCHD in Taipei City (Fig 1). Twelve birthing facilities participated, including 3 tertiary centers, 8 regional hospitals and 1 obstetrics and gynecology clinic, which represented 33% of the birthing facilities and 42% of all deliveries in Taipei during the study period. All newborns, including preterm babies, delivered in these facilities were eligible for inclusion in this study.

When admitted to the birthing facilities for childbirth, parents were informed that screening was available for CCHD. Of newborns who participate this screening, a verbal informed consent was obtained from the parents. If the parents refused to allow screening for CCHD, parents signed a written informed dissent. For health education and reinforcement, leaflets which listed the possible symptoms of CCHD and false-negative possibility were given to all parents. The unit cost of screening episode was $2.7 (USD), which was supported by the Department of Health, Taipei City government. The subsequent cost of diagnosis and treatment in screening positive cases were covered by the National Health Insurance in Taiwan.

All participating facilities were required to submit data on-line to database hosted by the TIP. This included the demographic data of each newborn, the test results and any referral information. To avoid artificial judgment mistakes, the result for the oxygen saturation values, which was stratified into “Pass”, “Rescreen”, or “Fail”) was determined automatically by the database on submission, according to a predefined screening criteria. The submitted demographic data (e.g., mother’s identification and the time of birth) was compared with that in the screening databases for congenital metabolic disease and hearing to identify missing cases daily. TIP monitored the databases daily and informed birthing facilities of missing cases or if the screening result had not been uploaded within 5 days after birth.

In Taiwan, all birthing facilities are required by law to report all live newborns within 7 days to the birth certificate registry system managed by the Ministry of Health and Welfare. The screening data were compared with the live birth data to calculate the coverage rate by the Department of Health of Taipei City. Data for newborns who were not screened were also collected. After cross-verification, public health nurses performed follow-up of the unscreened neonates, including missing and refused cases, within 1 month after delivery.

**Fig 2:**
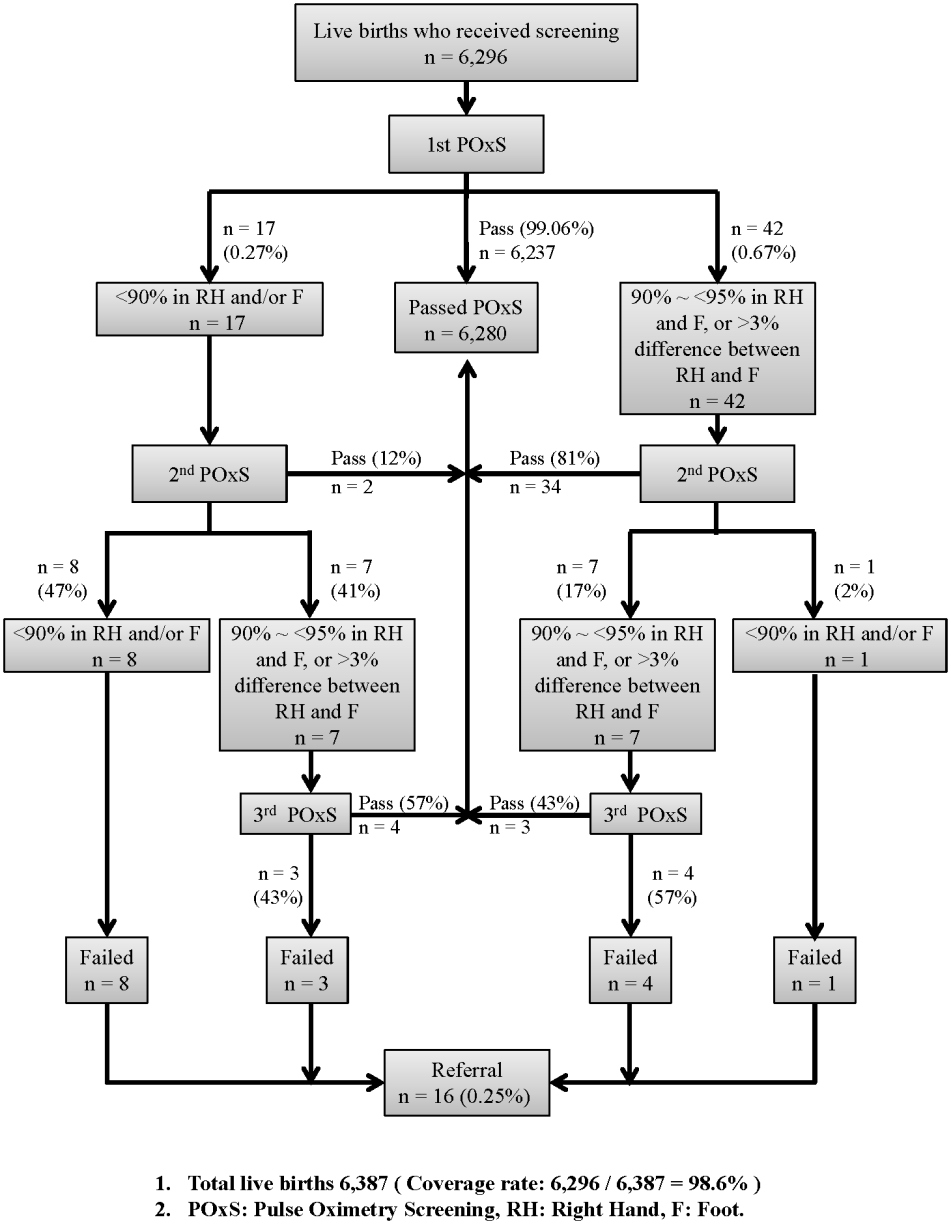
Aggregate pulse oximetry screening results from the 12 birthing facilities for live births. Total live births: 6,387 (coverage rate: 6,296/6,387 = 98.6%). Abbreviations: pulse oximetry saturation, POxS; right hand, RH; foot, F.

The screening algorithm (Fig 2) was modified from strategies proposed by a US working group (endorsed by the American Academy of Pediatrics, American College of Cardiology Foundation, and American Heart Association).[10] At the age between 24 and 36 h, pulse oximetry was performed by pediatricians or nurses experienced in the routine use of pulse oximetry. If the newborn discharged or referred to other hospital at <24 h of age, the screening performed before discharge or referral. The screening devices were required to have an in vitro diagnostic device registration permit from the Ministry of Health and Welfare, Taiwan and a ≤2% root-mean-square accuracy. Functional oxygen saturation was obtained from the right hand and from one foot (left or right). Any screening saturation ≧ 95% in either extremity with an absolute difference of ≤3% between the right hand and either foot, was accepted as a pass, and the screening ended. A screen result was considered as a fail if (1) the oxygen saturation was <95% at either measurement site on three separate occasions, each separated by 30 min or (2) the first screening result was <95% at either measurement site, and if any subsequent oxygen saturation measurement, performed at least 30 min after the first test, was <90%. These criteria applied to all live births regardless of health status and location (including neonatal intensive care unit, special care nursery, intermediate care nursery, or well-baby nursery). For those babies in supplemental oxygen, physicians-in-charge would determinate whether to hold oxygen supply for 10 mins and then screen, or to hold the screening till discharge from special care units. To assist with decision making at the bedside, all participating units were provided to a 2-way (hand and foot) decision chart and CCHD Screening Assistant App for Android and iOS mobile devices (http://cchd.pmf.tw/app/en/).

If a newborn failed the CCHD screening, an on-call pediatrician was required to perform clinical examination immediately, and the newborn was referred for urgent echocardiography. Of the 12 birthing facilities, 8 (67%) had pediatric on-site cardiologists and echocardiography facilities to provide confirmatory diagnosis. Two hospitals which were capable of confirming diagnosis and providing further medical and surgical treatment were used for referrals; screening-positive neonates born in facilities without on-site echocardiography were immediately referred to these 2 hospitals for diagnosis and treatment.

The primary or referral facility that confirmed the diagnosis was responsible for submitting the data for screening-positive infants to the CCHD database. Included in the confirmation report were the relevant prenatal diagnosis, findings of physical examination, and results of diagnostic evaluation (e.g., electrocardiography, chest radiography, echocardiography, and computed tomography) and managements. The process of referral and diagnosis was monitored by TIP and supervised by the Department of Health, Taipei City government. The database manager of TIP, who didn’t involve the research, anonymized and de-identified the participant information prior to analysis.

## Results

Over the six-month study period, 6387 live births were reported from the participating facilities. Of those eligible for screening,6,296 newborns (coverage rate: 6,296/6,387 = 98.6%) underwent pulse oximetry (Table 1). Infants were not screened if they died before screening (n = 21, 0.33%), parents refused screening (n = 28, 0.44%), or screening was missed (n = 42, 0.66%). Public health tracking confirmed that none of deaths before screening was caused by CCHD.

**Table 1:** Results of CCHD screening program in Taipei.

Period	Passed No. of screening					No. of Not-screened			Live birth No. ^5^
	1^st^	2^nd^	3^rd^	Referred	Total	Refused	Missed	Dead	
2013/10	1,060	8	2	1	1,071	7	11	3	1,092
2013/11	1,047	8	0	3	1,058	6	14	5	1,083
2013/12	1,016	6	2	4	1,028	6	3	5	1,042
2014/01	1,117	3	0	1	1,121	4	7	4	1,136
2014/02	931	2	0	2	935	1	4	0	940
2014/03	1,066	9	3	5	1,083	4	3	4	1,094
Total	6,237	36	7	16	6,296	28	42	21	6,387
(Ratio)	(99.06%)^1^	(0.57%)^1^	(0.11%)^1^	(0.25%)^2^	(98.6%)^3^	(0.44%)^4^	(0.66%)^4^	(0.33%)^4^	

^1.^ Ratio = Case number / Total screened number.

^2.^ Refer rate = Referred case number/ Total screened number.

^3.^ Coverage rate = Total screened number/ Live birth number.

^4.^ Ratio = Case number/ Live birth number.

^5.^ Live birth number based on Birth Certificate Registry System, Ministry of Health and Welfare, Taiwan.

Among the screened newborns, 59 cases (0.94%) needed a second oximetry measurement (Fig 2), of whom 28.8% (17/59) had oxygen saturations <90% at either site. Twenty-three cases (23/59 = 39%) failed the second measurement 30 min later, of whom 9 had oxygen saturations <90% at either site and were referred for further evaluation immediately. Fourteen infants then received a third oximetry measurement after another 30 min and half of them passed the test. Therefore, 16 newborns (0.25%) had a failed screening result.

The timing of screening is summarized in Table 2. Of those who underwent screening by pulse oximetry, 89.8% had a complete screen between 24 and 36 h after birth, 92.9% between 24 and 48 h, and 2.99% (n = 188) within 24 h. The median age at screening was 25 h. Health providers followed the outcomes of all 43 infants who passed their screening at the second or third measurements over a minimum duration of 7 months.

**Table 2:** Efficiency of CCHD screening in Taipei.

Period	Case No.		Age at screening					Age at referral			
	Screened	Referred	< 24 h	24-36h	37-48h	49-72h	73h-7d	< 24 h	24-36h	37-48h	49-72h
2013/10	1,071	1	39	937	45	39	11	1	0	0	0
2013/11	1,058	3	35	947	36	30	10	0	3	0	0
2013/12	1,028	4	26	954	23	23	2	1	1	2	0
2014/01	1,121	1	34	998	35	40	14	0	0	1	0
2014/02	935	2	28	832	27	35	13	0	1	1	0
2014/03	1,083	5	26	991	27	32	7	1	2	1	1
Total	6,296	16	188	5,659	193	199	57	3	7	5	1
(Ratio)		(0.25%)^1^	(2.99%)^2^	(89.88%)^2^	(3.07%)^2^	(3.16%)^2^	(0.91%)^2^	(18.75%)^3^	(43.75%)^3^	(31.25%)^3^	(6.25%)^3^

^1.^ Refer rate = Referred case number/Total screened number.

^2.^ Ratio = Case number /Total screened number.

^3.^ Ratio = Case number /Referred number.

All the 16 newborns with a failed screening result were referred for further evaluation, and 15 (93.8%) had a final diagnosis, within 72 h after birth (Table 2). The median age at diagnosis was 2 days. 5 cases had a proven diagnosis of CCHD (2 cases of d-TGA, 1 case of HLHS, 1 case of Ebstein anomaly, 1 case with DORV, single ventricle, and TAPVR)(Table 3). Of these 5 cases diagnosed with CCHD, 2 cases had antenatal diagnosis and symptoms as tachypnea and cyanosis immediately after birth; 1 case presented dyspnea and cyanosis before screening; the other 2 cases received further evaluation only because of their failed screening results. The diagnoses of the other 11 cases with a failed screening result included patent ductus arteriosus, transient tachypnea of the newborn, respiratory distress syndrome, and persistent pulmonary hypertension of the newborn (Table 3). Of these 11 cases, 8 cases needed further management, including oxygen therapy and ventilator support; 6 cases had symptoms of respiratory distress before screening; Near-half of them (5/11) were preterm babies. Thus, the false-positive rate of pulse oximetry screening for CCHD was 0.18%.

**Table 3:** Final diagnosis of CCHD screening referrals in Taipei.

Diagnostic Hospital	No. of referral	Final diagnosis			
		Normal	CCHD	Other cardiac problem	Respiratory problem
CR01	6	0	1	0	5
CR02	1	0	0	1	0
CR03	2	0	1	0	1
CR07	2	0	1	0	1
CR08	5	0	2	0	3
Total	16	0	5^1^	1^2^	10

^1.^ Including 2 TGA, 1 HLHS, 1 Ebstein anomaly, and 1 DORV with single ventricle and TAPVR.

^2.^ Patent ductus arteriosus.

## Discussion

In the present study, the case-referral and false-positive rates of pulse oximetry screening for CCHD were 0.25% and 0.18%, respectively. Because of different thresholds of positive test and screening results, the case-referral rate has been reported to be between 0.06% and 0.43%.[13, 18, 19] Previous studies have also reported that the false-positive rate varies between 0.026% and 1.8%,[13, 18] with a multicenter study from China reporting a 0.3% false-positive rate for CCHD.[19] In another research, Richmond *et al*. showed that repeat pulse oximetry brought their false-positive rate down from 5% to 1%.[20] Consistent with this, we found that the third measurement helped to reduce the false-positive rate and to diminish the cost of unnecessary referrals and examinations. Half (7/14) of those who needed a third measurement passed the screening finally.

The age of neonates at screening was another important factor affecting the false-positive rate. An obvious increase in the false-positive rate has been observed elsewhere when pulse oximetry screening was conducted before 24 h after birth,[9, 13] whereas Thangaratinam *et al*. have reported that false-positive rate decreased (from 0.5% to 0.05%) when pulse oximetry was postponed after 24 h from birth.[13] In a meta-analysis of 10 studies, comprising 123,846 newborns, the false positive rate was reported to be as high as 0.87% in the first 24 h after birth, but dropped to 0.035% if the newborns were screened after 24 h.[9] However, pulse oximetry screening within 24 h of age has an advantage to earlier detect and manage those hypoxic conditions resulting from non-cardiac lesions.[21] Even being taken as false positives for CCHD, most of these non-cardiac diseases still need special or intensive care.[22] In order to get balance between screening efficacy and medical resources utilization, this protocol designed newborn CCHD screening done between 24 and 36 h of age, which can be integrated into the screening window of the nationwide newborn hearing screening program in Taiwan.[17]

There were 2 important differences in the screening protocol between this study and the strategy proposed by the US working group.[10] The first modification was that newborns with a first screening result under 90% received a second measurement 30 min later; thus, in this study, these newborns were not immediately referred for further evaluation. The second modification was that we shortened the interval between measurements to 30min, which contrasted with the 1 h recommendation of the US working group.

In this study, 17 newborns had a first measurement <90%, of which 2 (12%) passed the oximetry screening at the second measurement. Of the remaining 15 newborns who failed the second screening measurement, 8 (47%) were immediately referred for further evaluation because their saturations remained <90%. The other 7 infants then received a third measurement, and 3 of them (18%) failed to achieve a screening pass. The true-positive rate was 65% (11/17) for a first screening measurement <90%, which meant that a second measurement for those neonates reduced referral rate by the one-third. None of these true-positive cases experienced any adverse events through repeat measurements from delaying referral by 30 min. In a prospective Swedish study, the authors reported that 22 of 24 normal babies with false-positive results had oxygen saturations <90% on the first measurement.[7] Under our protocol, a second measurement for those infants whose first measurement was <90% could improve the number of false positives and reduce the cost of further evaluation and referral. On the other hand, the shorter interval between measurements (30 min) appears to improve the likelihood that true-positive cases will get appropriate and expedient care.

All live newborns were eligible for this study, irrespective of gestational age or admission to a neonatal intensive care unit. From a public health perspective, newborns admitted to wards or intensive units of other diseases are still at risk of delayed detection of CCHD, especially in facilities without on-site echocardiography. The initial presentation of CHD could mimic sepsis, respiratory distress or other conditions.[23, 24] The symptoms due to progressive heart failure, such as tachypnea, sweating, feeding difficulty, and failure to thrive, are nonspecific.[25] Newborns with obstructive left heart disease may present with mottled gray skin, poor perfusion and decreased peripheral and central pulses. Some of these infants may be admitted with an initial impression of shock.[25, 26] Under these circumstances, pulse oximetry measurement of the right hand (preductal) and lower extremities (postductal) could be helpful.

Different from previous hospital-based programs, our newborn CCHD screening was implemented in a community-based system. This benefitted from the pre-existence of the electronic databases used in the screening of metabolic disease and hearing defects, which provided a readily available mechanism for incorporating individual-level screening results with clinical characteristics, details, and referral information in an established community reporting system. Furthermore, this allowed the tracking of eligible newborns, which helped to prevent missing cases. Manual interpretation of the data obtained from the right hand and one lower limb is relatively complicated. Indeed, experiences from an authoritative study suggest that efforts are needed to avoid the wrong interpretation and recording of screening results, such as those caused by human error.[4] In this program, we therefore used a 2-way chart and Tablet Personal Computer application to raise the accuracy of decision making at the bedside. The screening result was determined automatically according to the screening criteria upon submitting to the database. The reporting system we used also allowed real-time cross verification to prevent missing cases and included a method for referral follow-up. The network of public health nurses was responsible for following the outcomes of missing and refused cases. These coordinated measures helped to ensure the efficiency, accuracy and integrity of the CCHD screening database in this study.

This database lacking the outcomes of those newborns who pass the screening is a major limitation of this study. In our report, it was difficult to investigate the false-negative rate because of the impositions of the Personal Information Protection Act in Taiwan. However, the sensitivity of pulse oximetry screening has been reported to be 70% -75%,[11, 13] and the existence of false-negative cases is considered reasonable and unavoidable. Unfortunately there is no registry or notification system to detect false-negative cases in this screening program. For health education and reinforcement, all the parents of screened children were provided with a printed leaflet detailing the screening program and the significant abnormal symptoms and signs of CCHD. If any abnormal symptoms and signs noted, looking for medical service as soon as possible is suggested.

In conclusion, the results of this study suggest that pulse oximetry screening for CCHD could be successfully implemented as a part of the routine newborn screening program throughout Taiwan. Our study supports the arguments that performing a second measurement for neonates with initial oxygen saturations <90% and that repeating measurements at shorter intervals could lower the referral and false-positive rates, without delaying diagnosis and treatment. Our study reported 2 cases which had no prenatal detection and symptoms/signs before screening, detected solely by newborn CCHD screening program. This result supports that newborn CCHD screening program complements prenatal diagnosis and makes early detection before symptoms developed. Because of the successful experiences, the CCHD screening program has been implemented as a routine component of newborn screening in Taipei since April 2014.

## Abbreviations

CCHD: Critical congenital heart disease
HLHS: hypoplastic left heart syndrome
PS: pulmonary atresia
TOF: tetralogy of Fallot
TAPVR: total anomalous pulmonary venous return
d-TGA: d-transposition of the great arteries
TA: tricuspid atresia
CoA: coarctation of the aorta
DORV: double-outlet right ventricle
IAA: interruption of aortic arch
TIP: Taipei Institute of Pathology
